# Association between Peer Cigarette Smoking and Electronic Cigarette Smoking among Adolescent Nonsmokers: A National Representative Survey

**DOI:** 10.1371/journal.pone.0162557

**Published:** 2016-10-03

**Authors:** Jun Hyun Hwang, Soon-Woo Park

**Affiliations:** Department of Preventive Medicine, Catholic University of Daegu School of Medicine, Daegu, Republic of Korea; Leibniz Institute for Prvention Research and Epidemiology BIPS, GERMANY

## Abstract

We assessed the association between electronic cigarette (e-cigarette) use and peer cigarette smoking, a major risk factor for the initiation of cigarette smoking in adolescents. Data from the 2013 Korea Youth Risk Behavior Web-based Survey of 65,753 nonsmokers aged 13–18 years were analyzed using multiple logistic regression. A total of 3.8% of the Korean adolescents were ‘ever e-cigarette’ users and 1.2% were current users. Adjusted odds ratios (ORs) for current and ever e-cigarette use compared to those whose closest friends were non-smokers ranged from 2.05 (95% confidence interval [CI], 1.82–2.30) to 5.50 (95% CI, 4.77–6.34), and from 2.23 (95% CI, 1.77–2.81) to 7.82 (95% CI, 5.97–10.25) for those who had ‘some’ close friends to ‘most/all’ friends who smoked, respectively. The slopes of the adjusted ORs for e-cigarette use in ‘never smokers’ were more than twice as steep as those in ‘former smokers’, showing a significant interaction effect between the proportion of smoking closest friends and cigarette smoking status (never or former smokers) (p<0.001 for interaction). Peer cigarette smoking had a significant association with e-cigarette use in adolescent nonsmokers, and this association was greater on never smokers than former smokers.

## Introduction

Since the development of electronic cigarettes (e-cigarettes) in early 2000 and their introduction as an alternative to cigarettes, scientific evidences on their safety, smoking cessation effects, and adverse reactions have been accumulating [1–3]. Nevertheless, the e-cigarette market has grown rapidly through aggressive marketing using mass media such as the Internet [1,4–6]. In particular, e-cigarette companies are expanding the e-cigarette market primarily by targeting and marketing to adolescents and other young age groups through sponsorship of youth-oriented events, the development of e-cigarette flavors that appeal to youth, and lax youth e-cigarette purchase accessibility through online and offline stores [7,8].

According to the United States National Youth Tobacco Survey (NYTS), ‘ever e-cigarette’ use in high school students doubled in 1 year from 4.7% in 2011 to 10.0% in 2012 [9] while the estimated number of adolescent ever e-cigarettes users among ‘never cigarette smokers’ more than tripled from 79,000 in 2011 to 263,000 in 2013 [10]. McMillen *et al*. reported that the prevalence of current e-cigarette use in the 18–24 age group in the U.S. was 0.0% in 2010, but increased to 14.2% in 2013 [11]. Both the prevalence and margin of increase in the 18–24 age group were higher than for higher age groups. The 2013 Canadian Tobacco, Alcohol and Drugs Survey reported that younger age groups (15–24 years old) had higher current and ever e-cigarette use than older age groups (greater than 25 years old). These studies indicate that younger age groups have greater exposure to e-cigarettes [12].

Even if the continued debate about the efficacy of e-cigarettes as an alternative to cigarettes is excluded, the hypothesis that e-cigarettes are a gateway drug to cigarettes in nonsmokers has been proposed as a problem associated with e-cigarettes [5,13,14]. According to Bunnell *et al*. [10], intention to smoke cigarettes was 1.7 times higher in e-cigarette ever users than e-cigarette never users among adolescent never smokers. Dutra *et al*. [15] also reported that adolescent e-cigarette users showed high odds ratios (ORs) for current or ever cigarette smoking. According to Lee *et al*. [16], the OR of adolescent current e-cigarette users for current cigarette smokers was 64.9 times higher than that of e-cigarette never users. The NYTS study also supported the hypothesis [17] that e-cigarette ever users were more open to future cigarette smoking. Recent longitudinal studies in US and Swiss also supported that adolescent e-cigarette users were more likely to be cigarette smokers at follow-up assessments (6 or 12 month after baseline) [18–21]. Therefore, it is important to prevent adolescent never smokers from initiating e-cigarette use or former smokers from reinitiating cigarette smoking through e-cigarette use. To do so, it is necessary to identify the nonsmoker groups who are more likely to use e-cigarettes.

Given that friends’ cigarette smoking is a strong predictor of smoking initiation in adolescents [22–24], this peer influence is also suspected to significantly influence e-cigarette use in nonsmoking adolescents. However, there are few studies on this topic. To the best of our knowledge, the only cohort study on e-cigarette use related to peer group smoking is from Germany. This study reported that the OR of ever e-cigarette use in participants with smoker friends was 2.06 times (overall, including both former smokers and never smokers) or 1.78 times (never smokers) higher than in participants with no smoker friends [25]. In the U.S., a cross-sectional study of psychosocial factors for e-cigarette use showed that both e-cigarette and cigarette use among friends were strongly associated with current e-cigarette use in adolescents. The study showed that the ORs for current e-cigarette use increased with increasing number of friends who smoked tobacco products and ranged from 104 times (3 or 4 out of 4 closest friends smoked e-cigarettes) or 11.2 times (3 or 4 out of 4 closest friends smoked cigarettes) higher than those with no closest friends who smoked e-cigarettes or cigarettes [26]. However, the previous two studies were limited because they did not consider the effects of prior cigarette experience on e-cigarette use. Cigarette smoking status categorized as current, former or never smoker is not only closely associated with factors related to e-cigarette use but is in itself a strong risk factor for e-cigarette use [11,16,27]. Therefore, smoking status should be considered when analyzing factors related to e-cigarette use.

It is difficult to disentangle the impact of peer cigarette smoking and cigarette smoking status on youth e-cigarette use. In order to remedy this issue, and account for the effect of cigarette smoking status, it is necessary to restrict analyses of the impact of peer smoking on e-cigarette use to non-current smokers and to stratify these analyses into never and former cigarette smoking youth. Therefore, we used nationally representative data from Korean adolescents to investigate the association between peer cigarette smoking and e-cigarette use by nonsmokers and determine whether such association are also dependent on past cigarette experience.

## Materials and Methods

### Study population

Data were analyzed from the 10^th^ Korea Youth Risk Behavior Web-based Survey (KYRBS-X) conducted in 2014 by the Korea Centers for Disease Control and Prevention. The KYRBS is a nationally representative, self-reported, and anonymous online survey that was administered to Korean students enrolled in grades 7 to 12. The KYRBS uses a stratified multistage probability sampling design to produce nationally representative statistics on health behaviors in Korean adolescents. A total of 72,060 students from 799 schools (400 middle schools and 399 high schools) completed the KYRBS-X (response rate = 97.2%) [28]. Additional details about the sampling methodology and survey procedure are available elsewhere [29].After excluding current smokers, the study population included 65,753 nonsmokers (7,660 former smokers and 58,093 never smokers). Here, nonsmokers were defined as adolescents who reported not smoking in the past month. This secondary data analysis was exempt from review by the Institutional Review Board of the Daegu Catholic University Medical Center (CR-15-084).

### Measures

#### E-cigarette use

The e-cigarette use outcome variable was evaluated in two ways for current and ever e-cigarette use. First, ever e-cigarette use was defined as a “yes” response to the following question: “Have you ever used e-cigarettes?” Second, among ever smokers, participants who selected “yes” to the question “During the past 30 days, have you used e-cigarettes?” were considered current e-cigarette users.

#### Smoking-related factors

Peer cigarette smoking was assessed using responses to the following question: “Do any of your closest friends smoke tobacco?” Participants were provided with four possible answers: 1) None of them, 2) Some of them, 3) Most of them, and 4) All of them. Here, because the sample size of the ‘all’ group (0.8%) was too small to be separately categorized, this group and the ‘most’ group (5.0%) were combined (most/all). Cigarette smoking status among nonsmokers was classified into former smokers or never smokers using a composite measure of the two questions: “Have you ever tried cigarette smoking, even one or two puffs?” and “During the past 30 days, how many days did you smoke, even one puff?” Those who answered “yes” to the first question and “had not smoked in the past 30 days” to the second question were classified as former smokers. Those who answered “no” to the first question were classified as never smokers. Participants with a family member such as a parent, sibling, or grandparent who currently smoked cigarettes were considered to be living with a household member that used tobacco.

#### Other characteristics

Other covariates were categorized into two domains: sociodemographic and lifestyle. Sociodemographic variables included sex, school type (middle school, general high school, or vocational high school), region of residence (metropolitan city, city, or province), and perceived academic performance (high, middle, or low; classified using the question, “During the past 12 months, how would you rate your academic performance?”). Lifestyle and psychosocial factors included frequency of alcohol drinking per month (never, less than 6 times, or 6 or more times), experience of drug use (yes or no; classified using the question, Have you ever taken a drug or inhaled butane gas/bond habitually or intentionally), and perceived stress level (low, middle, or high; classified using the question, “How much stress do you usually feel?”).

### Statistical analysis

Multivariate logistic regression was conducted to estimate the relationship between peer cigarette smoking and e-cigarette use after adjusting for covariates including sex, school, location, perceived academic performance, alcohol intake, drug experience, perceived stress level, cigarette smoking status, and current smoking household member. In order to assess the association with peer cigarette smoking from the perspective of both simple smoking experience and current e-cigarette use, two different models were used to assess risk factors for current and ever e-cigarette use. Because cigarette smoking status is closely associated with e-cigarette use, an interaction model between peer cigarette smoking and cigarette smoking status was used to estimate the association between peer cigarette smoking and e-cigarette use according to cigarette smoking status. All analyses were performed using SPSS version 19.0 (IBM, Armonk, NY, USA) and a p-value of < 0.05 was considered significant. Complex SPSS sampling procedures were used to accurately represent the adolescent population in Korea.

## Results

Among adolescent nonsmokers, 3.8% and 1.2% were ever and current e-cigarette users, respectively. Ever e-cigarette use was significantly higher in former smokers (19.9% vs. 1.6% for never smokers; p<0.01) and in those with a current smoking household member (4.2% vs. 3.2%; p<0.01). Current e-cigarette use was also significantly higher in former smokers (6.3% vs. 0.5% in never smokers; p<0.01) and those with a current smoking household member (1.3% vs. 1.1%; p<0.05). The prevalence of ever or current e-cigarette use significantly increased as the proportion of closest friend smokers increased (p<0.01). Therefore, 19.8% or 8.7% of nonsmokers with most/all smoking closest friends were ever or current e-cigarette users, respectively. With the exception of location, all other well-known risk factors for cigarette smoking or e-cigarette use were significantly associated with e-cigarette use (Table 1).

**Table 1:** Summary statistics of variables by e-cigarette status among nonsmokers.

Characteristic	All^a^	E-cigarette use^b^		
		Never	Ever	Current
Respondents	65,753 (100.0)	63,384 (96.2)	2,369 (3.8)	752 (1.2)
Socio-demographic factors				
Sex				
Male	31,611 (49.4)	29,729 (93.8)	1,882 (6.2)^c^	634 (2.1)^c^
Female	34,142 (50.6)	33,655 (98.6)	487 (1.4)	118 (0.4)
School				
Middle school	34,543 (50.7)	33,720 (97.5)	823 (2.5)^c^	255 (0.8)^c^
General high school	26,592 (41.8)	25,353 (95.4)	1,239 (4.6)	380 (1.5)
Vocational high school	4,618 (7.5)	4,311 (92.6)	307 (7.4)	117 (2.7)
Location				
Metropolitan city	34,215 (52.2)	32,983 (96.3)	1,232 (3.7)	405 (1.3)
City	28,350 (43.9)	27,309 (96.1)	1,041 (3.9)	310 (1.2)
Province	3,188 (3.9)	3,092 (97.1)	96 (2.9)	37 (1.2)
Perceived academic performance				
High	25,307 (38.5)	24,586 (97.0)	721 (3.0)^c^	206 (0.9)^c^
Middle	18,755 (28.6)	18,182 (96.8)	573 (3.2)	187 (1.1)
Low	21,691 (33.0)	20,616 (94.8)	1,075 (5.2)	359 (1.8)
Lifestyle and psychosocial factors				
Frequency of alcohol drinking (per month)				
Never	58,276 (88.3)	56,735 (97.3)	1,541 (2.7)^c^	431 (0.8)^c^
<6	6,284 (9.8)	5,649 (89.6)	635 (10.4)	224 (3.6)
≥6	1,193 (1.9)	1,000 (82.8)	193 (17.2)	97 (8.9)
Experience of drug use				
Yes	371 (0.6)	286 (74.1)	85 (25.9)^c^	49 (15.3)^c^
No	65,382 (99.4)	63,098 (96.4)	2,284 (3.6)	703 (1.2)
Perceived stress level				
High	23,823 (36.2)	22,870 (95.8)	953 (4.2)^c^	290 (1.3)^c^
Middle	28,601 (43.7)	27,678 (96.7)	923 (3.3)	279 (1.0)
Low	13,329 (20.1)	12,836 (96.1)	493 (3.9)	183 (1.5)
Smoking-related factors				
Cigarette smoking status				
Former	7,660 (11.8)	6,187 (80.1)	1,473 (19.9)^c^	456 (6.3)^c^
Never	58,093 (88.2)	57,197 (98.4)	896 (1.6)	296 (0.5)
Closest friend smoking				
None	39,415 (58.4)	38,948 (98.8)	467 (1.2)^c^	119 (0.3)^c^
Some	22,778 (35.8)	21,574 (94.6)	1,204 (5.4)	328 (1.5)
Most/All	5,959 (5.8)	2,862 (80.2)	698 (19.8)	305 (8.7)
Household member current smoking				
Yes	38,550 (58.1)	36,995 (95.8)	1,555 (4.2)^c^	489 (1.3)^d^
No	27,203 (41.9)	26,389 (96.8)	814 (3.2)	263 (1.1)

Abbreviations: e-cigarette, electronic cigarette.

Data are presented as unweighted N (weighted percentage).

^a^Percentages are by column.

^b^Percentages are by row. Ever e-cigarette use indicates having ever tried an e-cigarette and current e-cigarette use indicates having used an e-cigarette in the past 30 days.

^c^p<0.01.

^d^p<0.05.

After adjusting for all of the covariates, former smokers were more likely to be ever (adjusted OR = 7.89; 95% confidence interval [CI], 7.17–8.69) or current e-cigarette users (adjusted OR = 5.32; 95% CI, 4.52–6.27) than never smokers. A dose-response relationship between the proportion of closest friend smokers and e-cigarette use was observed. Specifically, adjusted ORs for ever e-cigarette use increased from 2.05 (95% CI, 1.82–2.30) for participants with some closest friends who smoked to 5.50 (95% CI, 4.77–6.34) for those who reported most or all of their closest friends smoked. Similarly, adjusted ORs for current e-cigarette use increased from 2.23 (95% CI, 1.77–2.81) to 7.82 (95% CI, 5.97–10.25) across these groups (Table 2).

**Table 2:** Adjusted odds ratios* (95% confidence intervals) for current and ever e-cigarette use.

Characteristic	Dependent variables^a^	
	Ever e-cigarette use	Current e-cigarette use
Socio-demographic factors		
Sex		
Male	2.75 (2.44–3.10)	3.43 (2.76–4.26)
Female	Reference	Reference
School		
Middle school	Reference	Reference
General high school	1.17 (1.04–1.32)	1.06 (0.86–1.31)
Vocational high school	1.15 (0.94–1.41)	1.10 (0.79–1.53)
Location		
Metropolitan city	Reference	Reference
City	0.99 (0.89–1.11)	0.88 (0.72–1.07)
Province	0.75 (0.57–0.99)	1.02 (0.68–1.52)
Perceived academic performance		
High	Reference	Reference
Middle	0.97 (0.85–1.10)	1.12 (0.90–1.40)
Low	1.33 (1.19–1.49)	1.51 (1.25–1.82)
Lifestyle and psychosocial factors		
Frequency of alcohol drinking (per month)		
Never	Reference	Reference
<6	1.90 (1.69–2.13)	2.04 (1.72–2.42)
≥6	2.80 (2.27–3.45)	4.00 (3.0–5.25)
Experience of drug use		
Yes	5.76 (4.22–7.85)	8.12 (5.68–11.61)
No	Reference	Reference
Perceived stress level		
High	1.04 (0.91–1.19)	0.85 (0.69–1.05)
Middle	0.83 (0.74–0.95)	0.72 (0.59–0.88)
Low	Reference	Reference
Smoking-related factors		
Cigarette smoking status		
Former	7.89 (7.17–8.69)	5.32 (4.52–6.27)
Never	Reference	Reference
Closest friend smoking		
None	Reference	Reference
Some	2.05 (1.82–2.30)	2.23 (1.77–2.81)
Most/All	5.50 (4.77–6.34)	7.82 (5.97–10.25)
Household member current smoking		
Yes	1.15 (1.05–1.27)	1.03 (0.87–1.21)
No	Reference	Reference

*Adjusted for all covariates.

^a^ Reference group for calculating adjusted odds ratio is never e-cigarette use.

**Fig 1:**
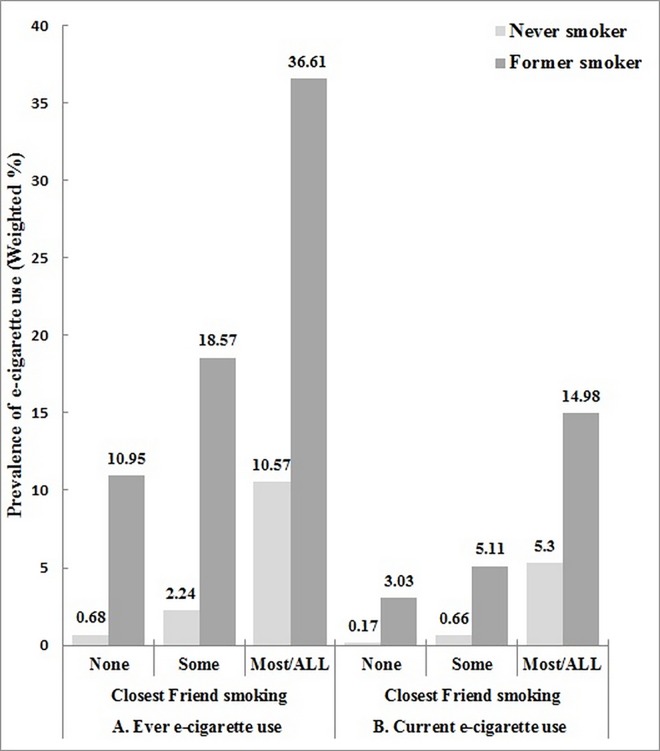
Prevalence of ever (A) or current (B) e-cigarette use according to the proportion of closest smoking friends among nonsmokers.

The prevalence of e-cigarette use increased steadily with an increase in the proportion of smoking closest friends among both never smokers and former smokers. Under the same condition of the proportion of smoking closest friends, the prevalence of ever or current e-cigarette use was consistently higher in former smokers than never smokers. However, the rate of increase of e-cigarette use prevalence in never smokers was greater than that in former smokers. In particular, while the prevalence of ever e-cigarette use in former smokers increased 3.3 times (from 10.95% in those with no smoking closest friends to 36.61% in those with most/all closest friends), it increased 15.5 times (from 0.68% to 10.57%) in never smokers (Fig 1).

**Fig 2:**
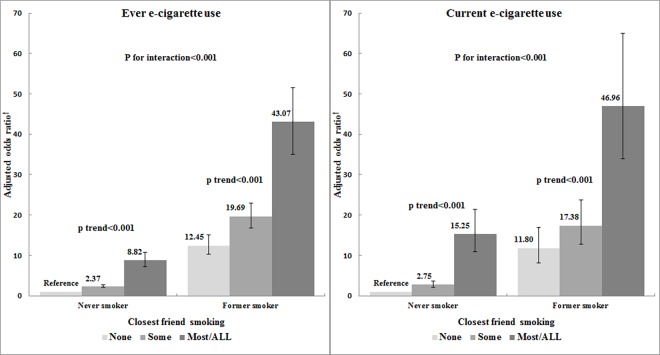
Adjusted odds ratios (95% confidence intervals) for e-cigarette use by the proportion of smoking closest friends and cigarette smoking status. ^†^ Adjusted for sex, school, location, perceived academic performance, alcohol intake, drug experience, perceived stress level, and household member current smoking.

The results from the interaction model between peer cigarette smoking and cigarette smoking status are shown in Fig 2. Regardless of cigarette smoking experience, the adjusted ORs for current or ever e-cigarette use increased significantly with increasing proportion of smoking closest friends in never smokers (p<0.001 for trend). This significant association was also present in former smokers. However, a significant interaction between the proportion of smoking closest friends and cigarette smoking status was observed in both ever and current e-cigarette use models (p< 0.001 for interaction) and the slopes of the adjusted ORs for current or ever e-cigarette use in never smokers were more than twice as steep as those in former smokers (Fig 2).

The adjusted ORs in interaction terms (Former ⨉ Some, Former ⨉ Most/All) were significantly lower than 1.0 in both the ever and current e-cigarette use models; this means that former smokers are less likely to be affected by the proportion of closest smoking friends compared to never smokers. The interaction effect was larger in the most/all group than the some group. Specifically, the adjusted ORs in interaction terms for Former ⨉ Most/All (ever e-cigarette use, 0.39; current e-cigarette use, 0.26) were lower than those for Former ⨉ Some (ever e-cigarette use, 0.69; current e-cigarette use, 0.54) (Table 3).

**Table 3:** Evaluation of the interaction effect between peer cigarette smoking and cigarette smoking status for current and ever e-cigarette use.

		Adjusted odds ratios (95% confidence intervals)*	
		Ever e-cigarette use	Current e-cigarette use
Closest friend smoking (A)	None	Reference	Reference
	Some	2.37 (2.03–2.75)	2.75 (2.04–3.70)
	Most/All	8.82 (7.27–10.70)	15.25 (10.83–21.48)
Cigarette smoking status (B)	Never	Reference	Reference
	Former	12.44 (10.21–15.16)	11.80 (8.18–17.02)
Interaction(A⨉B)	Former ⨉ Some	0.69 (0.53–0.85)	0.54 (0.35–0.82)
	Former ⨉ Most/All	0.39 (0.30–0.51)	0.26 (0.17–0.41)

*Adjusted for sex, school, location, perceived academic performance, alcohol intake, drug experience, perceived stress level, and current smoking household member.

## Discussion

This study showed that the proportion of closest friends who smoked had a significant relationship with e-cigarette use in adolescent nonsmokers. Both ever and current e-cigarette use increased significantly as the proportion of closest friend smokers increased, regardless of past smoking experience. This finding is consistent with results from a German cohort study that indicated peer cigarette smoking affected lifetime e-cigarette use [25]. Furthermore, our results suggest that in adolescents peer group cigarette smoking plays an important role in not only cigarette smoking but also in e-cigarette use. In particular, considering that the adjusted OR of peer group cigarette smoking was higher among the analyzed variables, the results were consistent with previous results that indicated peer group cigarette smoking can have a significant influence on various types of adolescent smoking such as smokeless tobacco [30, 31].

In a longitudinal study conducted with 12-year-old adolescents in the United States, as friend compliance (measured with the following question; “I do what my friends want me to do, even if I really don’t want to.”) increased, the use of smokeless tobacco also increased [32]. In addition, another cross-sectional study of U.S. adolescents showed that approval and use of e-cigarettes and cigarettes among friends were strongly associated with e-cigarette use [26]. These findings suggest that not only directly assessed peer smoking but also perceived peer influence or psychosocial factors regardless of cigarette use can influence the use of cigarette alternatives including e-cigarettes.

In addition to peer group cigarette smoking acting as a direct pathway to adolescent cigarette smoking, an alternative or indirect pathway to adolescent cigarette smoking can occur via e-cigarette use. Several longitudinal studies among adolescents or young adults in US or Swiss reported recently that e-cigarette use in adolescent non-smokers or never-smokers was closely associated with both willingness to smoke and smoking initiation [18–21]. Moreover, this phenomenon can be accelerated by the renormalization strategy from aggressive e-cigarette marketing.

In the past several decades, efforts have been made to establish desirable social norms about smoking through denormalization strategies, which are main strategies used in global tobacco control [33]. Tobacco industry denormalization strategies have also shown a reduction in the rate of adolescent cigarette smoking [34]. However, the psychological barriers to cigarette use formed through denormalization strategies has been threatened by e-cigarette company renormalization strategies through various marketing techniques [35]. As a result, there is the risk that lowered psychological barriers from renormalization strategies increase the likelihood of cigarette smoking via e-cigarette use (indirect pathway), rather than through peer group cigarette smoking (direct pathway).

In particular, the present study showed that the influence of peer group cigarette smoking on e-cigarette use was over 2 times higher in never smokers than former smokers and this difference could be explained by renormalization. In other words, psychological barriers to e-cigarette use have already been lowered in former smokers as a result of their past smoking experience. Consequently, former smokers may perceive e-cigarette use as non-deviant behavior; therefore, the role of renormalization strategies by e-cigarette companies may be nonsignificant in former smokers compared to never smokers. In contrast, in never smokers, the threshold of psychological barriers that recognizes e-cigarette use as deviant behavior may be lower than that for cigarette use due to renormalization strategies. As a result, it is believed that even never smokers who were not influenced by peer group cigarette smoking to initiate cigarette smoking may react to e-cigarette use and show a stronger response to peer influence than former smokers. Specifically, having more friends who were cigarette smokers was associated with a greater response margin to e-cigarette use regardless of past cigarette experience. Because having a greater number of peer group cigarette smokers leads to lower negative perceptions about cigarette smoking [36], these results suggest that the interaction between peer group cigarette smoking and e-cigarette renormalization had a more substantial influence on diminishing psychological barriers.

Because the present study was cross-sectional, it was unable to evaluate whether there was a causal relationship between peer group cigarette smoking and e-cigarette use. Moreover, because it posed no questions about social norms or perceptions about cigarettes or e-cigarettes, it was difficult to directly identify the cause of the differential influence of peer group cigarette smoking on e-cigarette use between former and never smokers. Thus, there is a need for continued study of this subject in the future. Despite these limitations, the present study had the following advantages. First, the study used nationally representative data from a large-scale survey including 2,369 ever e-cigarette users and 752 current e-cigarette users. Second, after excluding current smokers, analyses were performed by dividing the subjects into former and never smokers. This enabled accurate assessments of peer group cigarette smoking on e-cigarette use, including simultaneous analysis of the interaction between peer group cigarette smoking and cigarette smoking status. Finally, when comparisons were made with comparable 2011 U.S. NYTS data [15,16], it was revealed that e-cigarette use in Korean adolescents (4.7%) was more than 4 times higher than in U.S. adolescents (1.1%). Furthermore, among current smokers, the dual user rate (36.6%) was much higher than that of U.S. adolescents (10.6%). Moreover, the rate of current e-cigarette only users was higher in Korean adolescents (1.1%) than in U.S. adolescents (0.6%). Considering that e-cigarettes are widely marketed through the Internet and South Korea is globally an Internet powerhouse [37], South Korea is at risk for dramatic increases in future e-cigarette use. That being the case, the present study was meaningful in that it is the first to assess factors related to e-cigarette use in Korean adolescents.

## Conclusion

Peer group cigarette smoking had an important relationship with adolescent e-cigarette use and this relationship was greater on never smokers than former smokers. These findings give warning that peer group cigarette smoking can be combined with e-cigarette renormalization strategies to enable the expansion of the e-cigarette market by reaching adolescent never smokers who would otherwise not be interested in cigarette use. Not only did e-cigarette users have positive perceptions of e-cigarettes, they also had liberal views on future cigarette smoking and had high potential to become dual users [15–17,27,38]. Therefore, it is essential to continue to study e-cigarettes to accurately assess their health hazards and use e-cigarette denormalization strategies to instill proper e-cigarette perceptions in adolescents. This will mitigate adolescents’ expanded use of e-cigarettes and prevent cigarette smoking in this group.
